# The Contrived Mutant p53 Oncogene – Beyond Loss of Functions

**DOI:** 10.3389/fonc.2015.00276

**Published:** 2015-12-10

**Authors:** Kanaga Sabapathy

**Affiliations:** ^1^Division of Cellular and Molecular Research, National Cancer Centre Singapore, Humphrey Oei Institute of Cancer Research, Singapore; ^2^Cancer and Stem Cell Biology Program, Duke-NUS Graduate Medical School, Singapore; ^3^Institute of Molecular and Cellular Biology, Singapore; ^4^Department of Biochemistry, Yong Loo Lin School of Medicine, National University of Singapore, Singapore

**Keywords:** dominant-negative effect, gain-of-functions, mutant p53-addiction, oncogene, p53, tumor-suppressor

## Abstract

Mutations in *p53* are almost synonymous with cancer – be it susceptibility to the disease or response to treatment – and therefore, are a critical determinant of overall survival. As most of these mutations occur in the DNA-binding domain of p53, many of the clinical correlations with mutant p53 have been initially relegated to the loss of its transcription-dependent activities as a tumor suppressor. However, significant efforts over the last two decades have led to the vast knowledge on the potential functions of the mutated p53 protein, which have been attributed to the physical presence of the mutant protein rather than the loss of its wild-type (WT) functions. Beyond the inhibitory effects of mutant p53 on the remaining WT protein that leads to the dominant-negative effect in the heterozygous state, mutant p53’s presence has also been significantly attributed to novel gain-of-functions that lead to addiction of cancer cells to its presence for survival, as well as for their ability to invade and metastasize, elevating it to a contrived oncogene that drives the cancer cells forward. This review will summarize the functional consequences of the presence of mutant p53 protein on cellular and organismal physiology.

## Mutant p53

**Figure 1:**
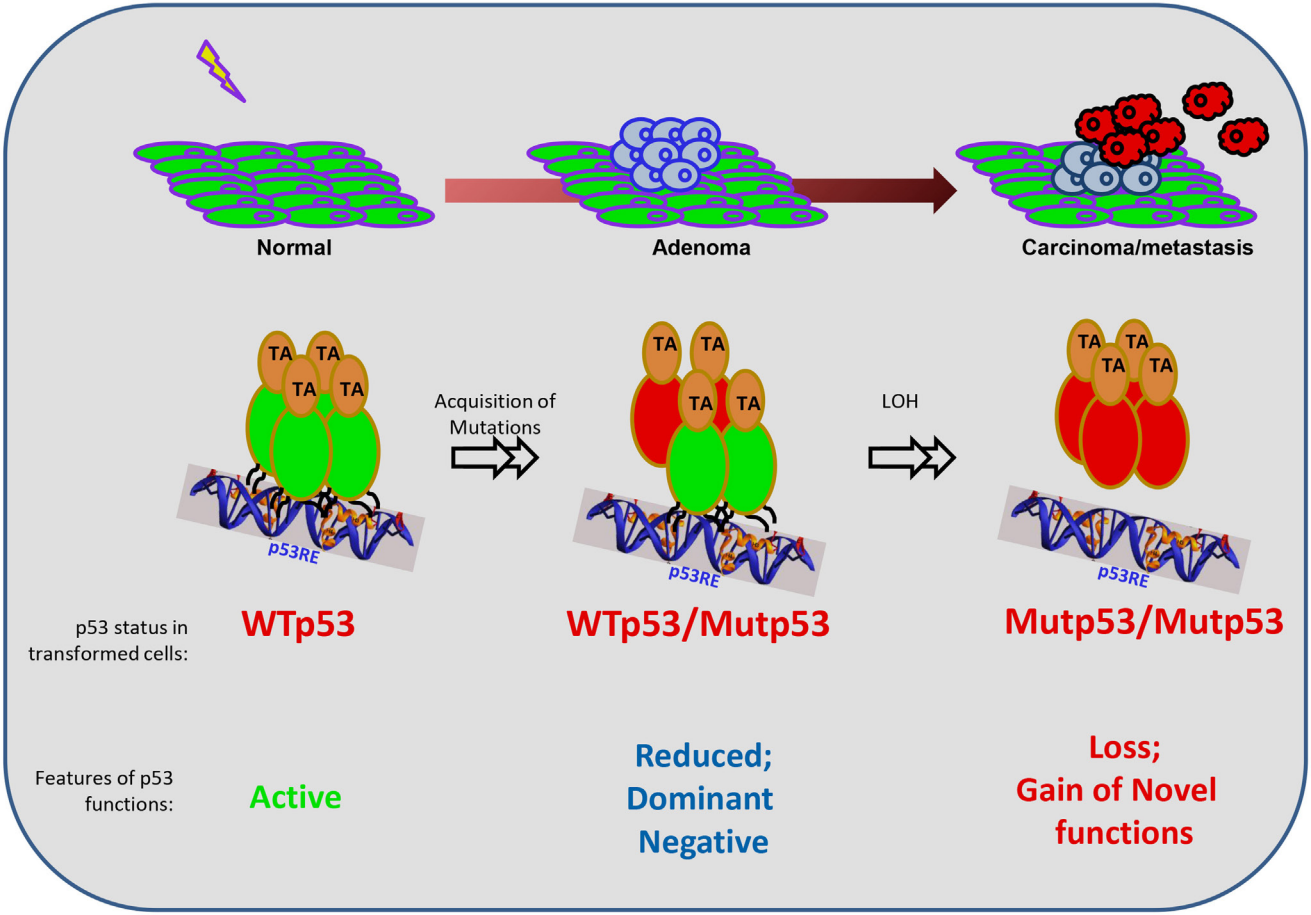
**Mutant p53 functions during the evolution of a cancer cell**. The schematic represents the general evolution of a normal cell into a transformed cell (carcinoma), and the contexts in which mutant (Mut) p53 exerts its functions. *p53* mutations are not present in the normal case and are induced upon genotoxic exposures in one allele. Hence, in the intermediate stage, the mutant p53 co-exists with the wild-type (WT) p53, until the loss of the wild-type allele by loss-of-heterozygozity (LOH). Functionally, when p53 is unmutated, it can be activated and works as a tetramer. However, when one allele is mutated, there is reduced overall function resulting in haploinsufficieny, and also the dominant-negative effect of the mutant protein on the wild-type protein due to the formation of heterodimers (please see text for details). At the later stages when only the mutant p53 remains, it is unable to bind to canonical target sequences to turn on its targets, leading to loss of wild-type functions. In addition, mutant p53 acquires novel gain-of-functions to drive the growth, survival, and invasion of tumor cells.

**Figure 2:**
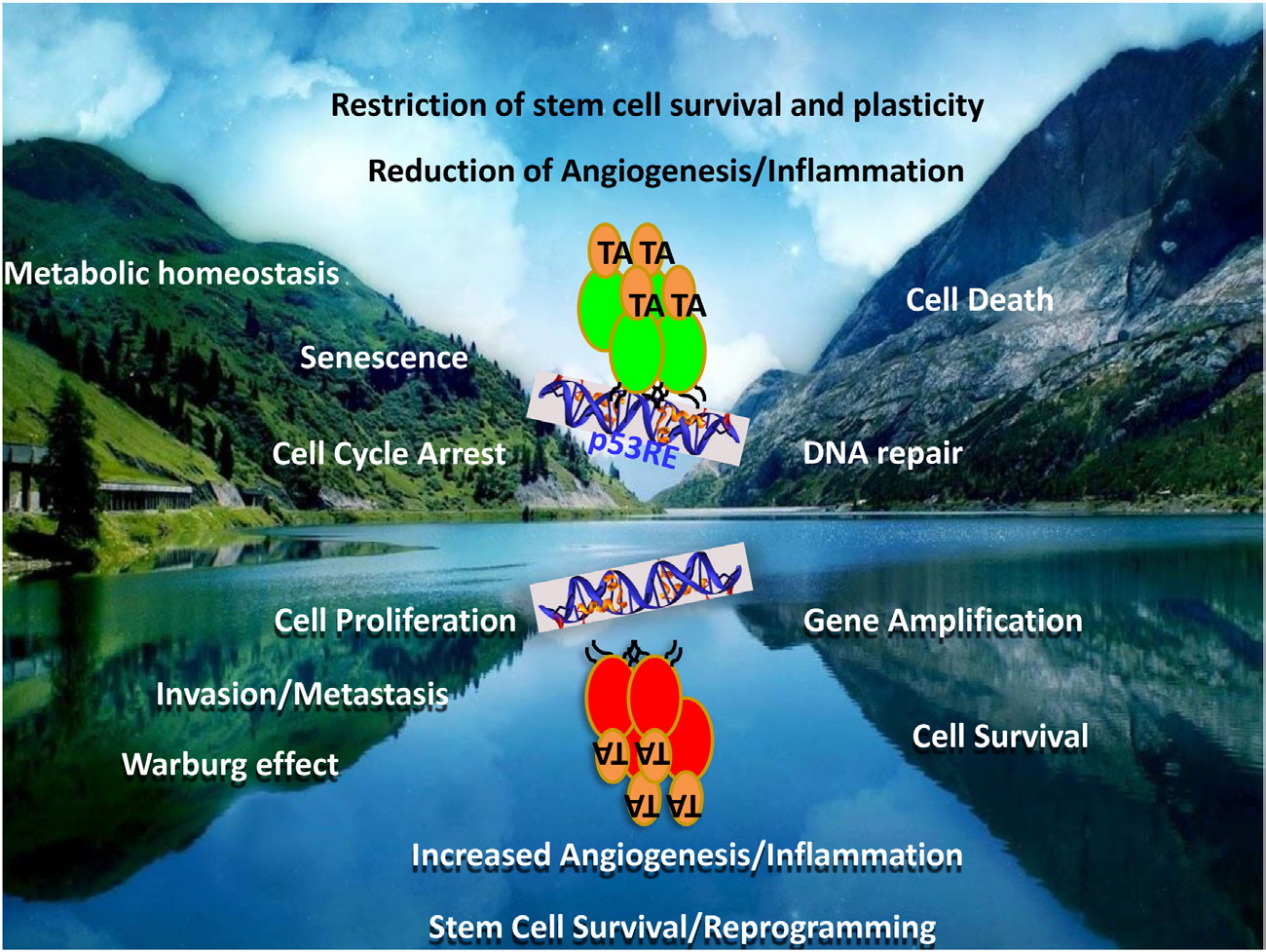
**Mutant p53 – the contrived oncogene**. The figure represents a mirror image of the functions of the wild-type and mutant p53 proteins. While wild-type p53 is a tumor suppressor, the mutant form represents not only a loss of these functions but also the acquisition of directly opposite functions. Many of the tumor-suppressor functions and the counteracting oncogenic functions by mutant p53 are represented as mirror-image pairs: cell death/cell survival; cell cycle arrest/cell proliferation; DNA-repair/genomic instability; senescence/invasion and metastasis; metabolic homeostasis/Warburg effect; restriction of angiogenesis and inflammation/increased angiogenesis and inflammation; restriction of stem cell plasticity and survival/increased reprograming and expansion, to highlight a few.

*p53*, one of the earliest identified tumor suppressors, has been overwhelmingly confirmed to be the most mutated gene across all cancer types through the recent avalanche of cancer genome sequencing efforts (1). Mutations in *p53* occur in about 90% of the cases in its central DNA-binding domain (DBD), thereby leading to loss of its transactivation properties that are often associated with its tumor-suppressor functions [reviewed in Ref. (2)]. During the course of the evolution of the transformed cell, mutant p53 derived from the mutated allele co-exists with the wild-type (WT) p53 from the other allele for varying time periods, till the WT allele is generally lost through loss-of-heterozygozity (LOH), resulting in the sole existence of only the mutant p53 (Figure 1). Like most of the tumor suppressors that have a direct impact on tumor growth upon their loss of function – thus qualifying them as tumor suppressors – mutations in *p53* were also thought to lead to loss of most of its tumor-suppressor functions that regulate almost all aspects of cellular physiology. Interestingly, during the co-existence phase of both the WT and mutant p53 proteins, haploinsufficiency leads to propensity for increased tumor development, as has been demonstrated in Li–Fraumeni patients as well as in a large number of model organisms expressing the mutant *p53* allele (3, 4). However, in contrast to most tumor suppressors, mutant p53 is unique in the sense that it has not only lost its tumor-suppressor functions, but evidence accumulating over the last two decades has assigned a wide variety of advantages to the cancer cell of having the mutant p53 protein. In short, the advantages of having a mutant p53 to the growth and survival of the tumor cells can be classified as a mirror image of the tumor-suppressor functions of WT p53 (Figure 2). Of interest is the fact that no other tumor suppressor has acquired such a wide array of novel functions as p53 when mutated, thereby elevating p53 to the status of a “contrived” oncogene, entirely on the basis that its functions as a tumor suppressor are turned on its head when the gene gets mutated. In this review, I will discuss the novel and acquired functions of mutant p53, specifically focusing on the DBD mutations.

## Dominant-Negative Functions of Mutant p53 – At the Early Stages

Generally, in the early phases of cancer development, mutated *p53* co-exists with the WT allele until the latter is lost due to LOH. In this co-existence phase, haploinsufficiency is a generally observed phenomenon associated with tumor development (5). However, effects of the mutant p53 over the WT’s regular functions – in a dominant-negative (DN) manner – have also been noted. Several early studies indirectly illustrated this DN effect, especially through the overexpression of the mutant p53 in WT p53 expressing cells, or vice versa. However, direct evidence for the DN effect of the mutant p53 protein on the WT p53 was shown in co-overexpression studies, demonstrating the quenching of WT p53’s ability to affect cellular transformation and transactivation of target genes, especially in transformed cell lines (6, 7). Co-expression of WT p53 with mutant p53 was also shown to affect the conformation of the former into a mutant conformation due to co-translation with the mutant form (8). Moreover, WT p53 was suggested to be inactivated through oligomerization with mutant p53 (9), and by inhibition of WT p53’s ability to bind to target gene promoters in a specific manner (6, 10). Concomitant to the effects on target gene activation, DN effects were noted on WT p53’s ability to induce cell death (11) and ras-induced senescence (12). Consequently, large efforts to analyze the DN effects of tumor-derived p53 mutants on the activation of several target genes have been undertaken in a systematic way using the yeast model, which was able to classify mutations as either dominant or recessive (13), and has also led to the cataloging of p53 mutations based on the ability to regulate a large number of p53-target genes (14).

Although the DN phenomenon has been well demonstrated in a large series of studies, the question that remained was its relevance when mutant p53 is expressed from its endogenous locus. This was resolved with the generation of the p53 mutant knock-in mouse models, whereby several groups confirmed the DN effects using p53^mutant/+^ mice in various primary cell types, including thymocytes, splenocytes, and embryonic stem cells, by comparing it with the p53^−/+^ cohorts (15–18). Interestingly, while the DN effects were seen in some tissues, both on target gene activation and on cell survival, this was not the case in other cell types, as in primary fibroblasts in their growth, suggesting that the DN effect might be cell type, and possible stimulus dependent (18).

An interesting observation that emerges from all these studies with primary and transformed cells is that the DN effects on target gene activation and cell death are generally seen when cells are exposed to stress stimuli, including exposure to DNA-damaging agents, when p53 is activated and stabilized (15, 16, 18). By contrast, DN effect is not normally observed at baseline conditions, as there were no growth advantages to primary cells from p53^mutant/+^ mice, or with respect to spontaneous tumor development, both of which mirrors the p53^−/+^ cells and mice (18, 19). Furthermore, although the DN effect can lead to almost complete ablation of target gene activation when observed, all p53^mutant/+^ mice generated so far have not been able to rescue the embryonic lethality due to the absence of Mdm2 (15, 18). Thus, these data collectively surmise that the DN effect of mutant p53 is exhibited when the levels of the mutant p53 is elevated in acute stress conditions, and thus, may have a significant consequence when patients are treated with chemotherapy and radiotherapy. However, a noteworthy point is that DN effects are not seen with respect to susceptibility to tumor formation, even in the case when p53^mutant/+^ mice have been irradiated, supporting the notion that DN effects observed upon acute activation of p53 that affects short-term apoptotic response do not have a contributory role to the long-term tumorigenic effects (18). Thus, acute p53 activation and DN effects of mutant p53 can be decoupled from the long-term effects of p53 in regulating tumor susceptibility.

In this respect, early reports have suggested that at least three molecules of mutant p53 are required to impose a DN effect on one molecule of WT p53 (7, 20). Interestingly, although mutant p53 protein has been shown to have a much longer-half life than WT p53, especially in tumor cell lines and in transfection studies, primary cells and tissues from p53^mutant/+^ mice do not exhibit elevated steady-state levels of the mutant p53 protein (18, 19). However, mutant p53 appears to be more abundant in tumor tissues from these mice, further alluding to the requirement of stress and/or oncogenic signals for mutant p53 stabilization (18, 19). Given that stress signals are also able to further stabilize mutant p53, it is not unexpected that the ratio of mutant p53 to WT is significantly high, thereby leading to the observed DN effects. In supporting this theory, reduction of the endogenous mutant p53 levels – using an hypomorphic mutant p53 knock-in mouse model – was indeed shown to alleviate the DN effects, both on target gene activation and cell death upon irradiation (18). Similarly, reduction of the WT p53 levels in a p53^mutant/+^ mice strain also led to exhibition of the DN effect in tumors (21). Thus, these observations consolidate the case for the requirement of a significant increase in mutant p53 levels – as seen in tumor cells, or in primary cells upon exposure to stress stimuli – for the manifestation of the DN effects, which could be ameliorated by reducing the mutant protein levels. This implies that in a clinical setting, strategies that would reduce mutant p53 protein levels without an effect on WT p53 during therapy would lead to better efficacy of treatment and would be a future prospect that should be followed up.

Mechanistically, there are a few modes of operation of the DN effect. The mutant p53, which itself is unable to specifically bind to the p53-response elements, binds to the WT p53, thereby quenching it away from target gene promoters (6, 10). Alternatively, mutant p53 quenches away co-factors that are required for transactivation by the WT p53 bound to the promoter, thereby reducing the potency of the WT protein (22). In addition, mutant p53 has been suggested to form aggregates, akin to those seen in protein misfolding diseases. Herein, it has been suggested that the WT p53 protein is engulfed into mutant p53 fibrillar and granular aggregates, whereby the misfolded mutant protein sequesters the WT form, thus leading to inactivation of the WT function (23). Whatever the mechanism may be, the underlying concept is that the ability of mutant p53 to bind to wild-p53 when in excess is causal to the DN effect, which could thus offer an avenue for exploitation for therapeutic benefit. While there are currently no known ways of overcoming the DN effect, potential strategies that lead to the degradation of mutant p53 specifically without affecting the WT protein will be the way forward in ameliorating the DN effect.

## After LOH – The Mutant p53-Addiciton Phenomenon

While the phenomenon of addiction to oncogenes has been well established (24), mutated tumor suppressors have never been earlier reported to provide a survival advantage to tumor cells due to any novel acquired functions. However, two reports in the mid-2000s showed for the first time that silencing the expression of endogenous mutant p53 can lead to increased apoptosis (25), and reduced tumor growth *in vivo* (26), formally demonstrating the phenomenon of addiction of tumor cells to mutant p53’s presence. In addition, indirect evidence for the requirement for mutant p53 for survival of cells in a phospholipase D-dependent manner was also noted (27). This phenomenon is now well established in a large number of subsequent studies. However, the causal mechanisms are still relatively elusive. In earlier studies, a role for transactivation by mutant p53 of cell growth regulation genes was suggested, as the transactivation deficient DBD p53 mutants were unable to provide a growth advantage (28). In the other studies, mutant p53-mediated suppression of canonical p53-target genes was suggested to be the mechanism, which was ameliorated upon the silencing of mutant p53 expression, leading to cell death. In this latter case, hypomethylation appeared to be involved, as trichostatin A – a HDAC inhibitor – was found to relief the mutant p53-dependent suppression (25). Other recent studies have confirmed this possibility, using other HDAC inhibitors, such as SAHA (29) and sodium butyrate (30).

Recent *in vivo* work in mice has also confirmed that destabilization of mutant p53 expression leads to apoptosis and reduction of tumor growth (31). In this case, mutant p53 was destabilized through the inhibition of the HSP90/HDAC6 chaperone machinery that is often upregulated in cancers, collectively highlighting the mutant p53-addiction phenomenon, and that degradation of mutant p53 can indeed enhance tumor cell death and improve therapy. While addiction to mutant p53 appears to be critical for the survival of the cancer cell, the exact point at which they get addicted to mutant p53 is not understood. While the transformed cells could be expected to become addicted to the presence of mutant p53 at the point in time of loss of the remaining WT p53 allele, it is likely that further events are required for this phenomenon to occur. Moreover, whether addiction to mutant p53 is a universal phenomenon in all cell types also requires further systematic analyses.

## Role Reversal from Tumor Suppressor to Oncogene: Gain of Novel Functions

Similar to mutant p53 addiction, the direct advantages conferred by the presence of the mutant p53 protein have been understood primarily through cell culture studies where isogenic cell lines without p53 expression have been used to analyze the effects of the overexpressed mutant p53. These have resulted in the uncovering of a plethora of gain-of-function (GOF) effects, almost all of which provide survival/growth advantage to the cell. First direct evidence came from the overexpression of several human (e.g., R175H, R248W, R273H, and R281G) and mouse mutants in p53 null cell lines that lead to increased cellular growth in soft agar and increased tumorigenicity in immunocompromised mice (32). While this was the first case of evaluating the effects of the mutant version of the natural tumor suppressor, earlier studies using a mutant p53 – at that time thought to be the natural existing form prior to the knowledge that p53 is actually a tumor suppressor – also showed growth advantage due to its overexpression (33). In addition, there are multiple other parameters associated with genomic instability that were noted due to the overexpression of mutant p53, including gene and centrosome amplification and disruption of spindle checkpoint (34–37). Consistently, overexpression of mutant p53 also led to resistance to death induced by a variety of chemotherapeutic drugs and DNA-damaging agents (38–41), as well as by anoikis (42). More recently, expression of mutant p53 was also shown to enhance the Warburg effect on cancer cells, promoting GLUT1 translocation to the plasma membrane, and thus enhancing glucose uptake (43).

Not unexpectedly, many studies have also evaluated if the expression of mutant p53 would enhance cellular invasion and migration and found that to be the case in a variety of 2D and 3D cellular systems (18, 44–46). Furthermore, a role for mutant p53 in promoting cellular reprograming was also demonstrated (47), suggesting that mutant p53’s presence would lead to the survival and replenishment of the potential cancer stem cells, leading to their ability to colonize the adjacent territory.

At the organismal level where mutant p53 is expressed from its own locus – reflecting the status in human cancer conditions – GOF properties were also noted with the generation of the p53 mutant knock-in mice. The initial data demonstrated that the p53^R172H^ mice (equivalent to human R175H) were more tumor prone with more carcinomas – reflecting a change in tumor spectrum. They also had increased metastasis compared to the p53 null mice, in the absence of Mdm2, which leads to increased mutant p53 levels (15, 16). Further studies have cemented these findings, where the presence of the R172H mutant p53 protein conferred significant growth and metastatic propensity to tumors compared to the loss of p53, in several oncogene-induced models, including Ras and APC, in a variety of tumor types such as lung and pancreatic ductal adenocarcinomas (46, 48, 49). Similar observations were also noted with other mutations, such as the R270H and R273H in breast and lung cancer models (50, 51). These data mooted the idea that all mutant p53 would have GOF properties, and that this may depend on the stabilization of the mutant p53 in the cancer cell context, as normal untransformed tissues from the mutant p53 bearing mice did not have significant growth advantage and did not display increased steady-state levels of the mutant protein (19). However, this theory came under challenge through the analyses of another hot-spot p53 mutant knock-in mouse strain (the R246S, equivalent to human R249S). In this case, the R246S mutant mice did not display any tumor latency difference or metastatic advantage even in the absence of Mdm2 (18). Similar results were also seen with other hot-spot mutant knock-in mice strains, in which the R248Q had a strong GOF phenotype in contrast to the G245S mutant (52), collectively alluding the fact that GOF may not be a universal phenomenon, and that elevation of mutant p53 levels may not be sufficient for their exhibition.

Nonetheless, as observed in the cell culture-based studies, there was a propensity to have more hematopoitic or mesenchymal stem cells in the R248Q mutant p53 knock-in mice, indicative of an effect of mutant p53 on the plasticity of the stem cell population (52). Besides the effects on tumor metastasis and aggressiveness, the effects of mutant p53 on several other aspects of physiology have been noted using the knock-in mice strains. For instance, there was increased inflammation and tissue damage, primarily due to upregulation of inflammatory cytokines that were induced by mutant p53-mediated prolonged activation of NFKB signaling (53). Angiogenesis was also shown to be enhanced due to mutant p53 expression, through the activation of ID4 expression in cell culture studies (54). These studies collectively indicate that expression of mutant p53 in tumor cells, as seen in the case with human cancers, as well as in normal tissues as analyzed from animal studies, has far-reaching consequences on organimsal physiology.

While enormous amounts of data from cellular systems and animal models highlight the existence of GOFs of mutant p53, albeit to varying degrees perhaps depending on several contextual factors, the main question is the relevance of this phenomenon in humans. A noteworthy point is that humans generally do not carry a mutant *p53* allele in non-transformed tissues, except in the case of the Li–Fraumeni patients. Thus, the GOF properties would be of relevance in the large majority of tumors that eventually retain only the mutant allele. On the other hand, the Li–Fraumeni patients would be expected to have one allele that is WT in all untransformed tissues, except in cases where LOH would result in or occur with other transforming events that lead to tumorigenesis. In this context, transcriptomic analyses of p53^−/−^ vs. p53^mutant/mutant^ primary tissues (e.g., thymus) from the knock-in mice remarkably did not reveal any significant changes (18), highlighting that GOF is generally not observed in untransformed cellular contexts. Thus, even in the Li–Fraumeni group, the GOFs and addiction to mutant p53 would be a phenomenon that would be of relevance primarily in the cancer cell context. This has been exemplified in a large number of studies that have evaluated the role of mutant p53 in response to chemotherapy, which have generally shown poor prognosis associated with the presence of mutant p53 (55). This, however, could either be due to the DN effect or the GOF functions, as many of these reports have not teased out the status of the other allele in the patient samples. Thus, the presence of mutant p53 in human tumors does indeed exert a negative effect in response to therapy, warranting in-depth investigations into the mechanisms of actions of mutant p53.

## Mechanisms and Mediators of Mutant p53 Gain-of-Functions

Since the demonstration of GOF of mutant p53, extensive efforts have gone on to examine the mechanistic basis of this phenomenon [reviewed in Ref. (56)]. At least four inter-related categories of actions have emerged: novel target gene activation through direct DNA binding; enhancement of target gene activation due to co-factor binding to mutant p53; co-operation with other transcription factors to activate other transcriptomes; and binding to factors that indirectly result in activation of other pathways.

In the first instance, while DBD-mutant p53 has lost its abilities to bind to canonical p53-responsive elements on target gene promoters, several studies have alluded to the ability of mutant p53 to bind to novel target gene promoters to activate them. It has been proposed that mutant p53 interacts with matrix-attachment regions (MARs), thereby recognizing structures rather than sequences (57). This results in the activation or repression of mutant p53-target genes, the list of which has been steadily growing. In brief, the targets can be categorized into either genes that are upregulated or down-regulated by the presence of mutant p53, including both coding and non-coding genes involved in promoting proliferation, inhibiting cell death, promoting migration, and inflammation. In the former category exists a large array of genes, including growth factors/receptors, such as bFgF, Egfr, Igf1, Igf1r, IL6, and TNFα; cell death/survival genes, such as bcl-xl, procaspase 3; oncogenes and transcription factors, such as c-fos; c-Myc, Egr1, Nfkb, Ras, and Egr1; metastasis regulators, such as twist-1; microRNAs, such as mir 155 and mir128-2; and many others [reviewed in Ref. (2)]. The second category of suppressed genes includes Fas, Mst-1/msp, mir130b, mir27a, mir 223, and also a large array of the canonical p53-target genes that appear to be suppressed by the presence of mutant p53 (25).

Next, regulation of target genes by mutant p53 can be facilitated by co-operative binding and post-translational modifications of the mutant p53 protein. For instance, mutant p53’s activity has been shown to be enhanced by binding with PML and Pin1 (58, 59). In addition, mutant p53 has also been shown to co-operate with other transcription factors, such as NF-Y, VDR, Sp1, and SREBP, to enhance the activation of their target genes (60–63). Conversely, mutant p53 has also been shown to bind to p63 and p73 to inhibit the activation of the latter groups’ targets (64). Finally, an indirect role for mutant p53 in activation of various pathways has emerged. For example, mutant p53 has been shown to bind to p63, thereby negating the latter’s inhibitory effects on the α5β1/RCP complex, which leads to enhanced cellular motility (46). Similarly, mutant p53 has been shown to bind to proteins in the DNA-repair pathway, such as MRE11, thereby affecting their functions, which leads to increased genomic instability (65).

The diversity in mechanisms and mediators of mutant p53’s GOF function allude to the fact that these may be dependent and vary according to the cell type, the mutation type or the stimuli. Furthermore, GOFs would also likely be temporally dictated during the evolution of the transformed cell. Hence, efforts aimed at targeting mutant p53 would have to take into account these factors that have to be elucidated and characterized.

## Future of Mutant p53

One can envisage that all the years of work on mutant p53 and its functions will now put the research community in good stead in trying to target its functions for clinical benefit, and the current status of these efforts is reviewed in the adjoining chapters. Nonetheless, important considerations have yet to be worked out. These include several questions, such as how can the DN effect be mitigated when treating patients whose tumors retain the WT allele; what is the effect of the DN phenomenon in the daily lives of LF patients, especially when they are exposed to a variety of signals that may have an acute effect on p53 activation; precisely when and where is GOF manifested and addiction to mutant p53 occur; and what is the trigger point for GOF of mutant p53. Thus, while general targeting strategies are being worked out, more work is required to realize the dream of targeting all types of mutant p53 that are different, and thus, likely require specific molecules/dugs to counter them.

## Author Contributions

KS planned and wrote the manuscript.

## Conflict of Interest Statement

The author declares that the research was conducted in the absence of any commercial or financial relationships that could be construed as a potential conflict of interest.
