# Corrigendum: The Contrived Mutant p53 Oncogene – Beyond Loss of Functions

**DOI:** 10.3389/fonc.2021.665504

**Published:** 2021-03-31

**Authors:** Kanaga Sabapathy

**Affiliations:** ^1^Division of Cellular and Molecular Research, National Cancer Centre Singapore, Humphrey Oei Institute of Cancer Research, Singapore; ^2^Cancer and Stem Cell Biology Program, Duke-NUS Graduate Medical School, Singapore; ^3^Institute of Molecular and Cellular Biology, Singapore; ^4^Department of Biochemistry, Yong Loo Lin School of Medicine, National University of Singapore, Singapore

**Keywords:** dominant-negative effect, gain-of-functions, mutant p53-addiction, oncogene, p53, tumor-suppressor

In the original article, we overlooked to acknowledge the funder **National Research Foundation, Singapore**, **NRF-NRFI-2015-007** to **Kanaga Sabapathy**.

The authors apologize for this error and state that this does not change the scientific conclusions of the article in any way.

